# Wearable Inertial Measurement Unit to Accelerometer-Based Training Monotony and Strain during a Soccer Season: A within-Group Study for Starters and Non-Starters

**DOI:** 10.3390/ijerph18158007

**Published:** 2021-07-28

**Authors:** Hadi Nobari, Mustafa Sögüt, Rafael Oliveira, Jorge Pérez-Gómez, Katsuhiko Suzuki, Hassane Zouhal

**Affiliations:** 1 Department of Physical Education and Sports, University of Granada, 18010 Granada, Spain; 2 HEME Research Group, Faculty of Sport Sciences, University of Extremadura, 10003 Cáceres, Spain; 3 Department of Exercise Physiology, Faculty of Sport Sciences, University of Isfahan, Isfahan 81746-7344, Iran; 4 Sports Scientist, Sepahan Football Club, Isfahan 81887-78473, Iran; 5 Department of Physical Education and Sports, Faculty of Education, Middle East Technical University, 06800 Ankara, Turkey; msogut@metu.edu.tr; 6 ESDRM-IPS-Sports Science School of Rio Maior—Polytechnic Institute of Santarém, 2040-413 Rio Maior, Portugal; rafaeloliveira@esdrm.ipsantarem.pt; 7 Research Center in Sport Sciences, Health Sciences and Human Development, Quinta de Prados, Edifício Ciências de Desporto, 5001-801 Vila Real, Portugal; 8 Life Quality Research Centre, Complexo Andaluz, Apartado, 2040-413 Rio Maior, Portugal; 9 Faculty of Sport Sciences, Waseda University, Tokorozawa 359-1192, Japan; 10 Movement, Sport, Health and Sciences Laboratory (M2S), University of Rennes 2, F-35000 Rennes, France

**Keywords:** football, external training load, WIMU, GPS, acceleration, deceleration

## Abstract

The purpose of this study was to analyze the intragroup differences in weekly training monotony (TM) and training strain (TS) between starter and non-starter male professional soccer players at accelerometry based variables throughout the periods of a season. TM and TS of different accelerations and decelerations zones for twenty-one players were followed for forty-eight weeks. Regardless of group, players obtained the highest mean TM (starters = 3.3 ± 0.6, non-starters = 2.2 ± 1.1, in arbitrary unit, AU) and TS (starters = 1288.9 ± 265.2, non-starters = 765.4 ± 547.5, AU) scores in the pre-season for accelerations at Zone 1 (<2 m/s^2^). The results also indicated that both groups exhibited similar TM and TS scores in accelerations at Zones 2 (2 to 4 m/s^2^) and 3 (>4 m/s^2^) across the entire season. While the starters showed the highest TM and TS scores at deceleration Zone 1 (<−2 m/s^2^) in the end-season, the non-starters exhibited the highest scores at the deceleration Zone 1 in pre-season. It seems that in pre-season, coaches applied higher levels of training with greater emphasis on deceleration for non-starters. This tendency was reduced over time for non-starters, while starters presented higher values of deceleration Zone 1. These results highlight the variations in TM and TS across the different periods of a full season according to match starting status among professional soccer players, and the results suggest that non-starter players should receive higher levels of load to compensate for non-participation in matches throughout a soccer season.

## 1. Introduction

Monitoring training load in professional soccer is one of the primary focuses of coaching staff in order to analyze individual training requirements, to optimize physical fitness, and to minimize the risk of injury [1,2]. The process of training load monitoring can be categorized as either internal or external with respect to the load units [3]. Internal training load is related to the psychophysiological stress imposed on players during training and competition, and it is usually measured using ratings of perceived exertion, blood lactate, oxygen consumption, and heart rate. External training load includes measures of training load data derived from advanced technological devices such as global positioning system (GPS) and electronic tracking systems [4].

Other commonly used training load measures are training monotony (TM) and training strain (TS). The TM is a measure of day-to-day variations in training load, and it is calculated as the mean daily training load divided by the weekly standard deviation load, while TS is a product of TM and weekly training load [5]. More specifically, rather than performing an equal daily training load throughout the week, interspersing low and high loading days can help maintain lower or moderate monotony and strain [6]. Furthermore, quantifying weekly TM and TS may prevent overtraining syndrome and negative health consequences [7,8].

Previous studies have documented the seasonal changes in training load, TM, and TS in professional and collegiate soccer players [9,10,11,12]. Fessi et al. [11] revealed that exposure to higher pre-season training loads resulted in higher TM, TS, and a lower psychophysical state during the in-season. In other recent studies, match starting status-related differences in accumulated workload, TM, and TS were investigated, and greater values were reported for starting soccer players compared to their non-starting counterparts throughout the season [10,13]. The discrepancy in accumulated training and match workloads between starter and non-starter players may also have consequences on physical fitness levels. The findings of the several earlier examinations demonstrated the associations of playing time status with seasonal changes in body fat percentages, sprinting ability, and muscular strength capacity [14,15]. For example, a significant increase in body fat percentage was reported for non-starters, while significant decrements were found in sprinting ability and vertical jump performance for starters throughout a season [14].

Nevertheless, there is scarce information regarding the intragroup differences in starting and non-starting soccer players with respect to the weekly TM and TS at different speed zones and across the periods of a full season. Recently, and in opposition to the previous information, the study from Oliveira et al. [16] found that both starters and non-starters presented small differences. The same authors suggested that training workload adjustments applied over the season helped to reduce differences between player status.

Therefore, the purpose of this study was to determine the within-group variations in TM and TS at different speed zones during a professional soccer season in both starters and non-starters. The present study is a complement to a previous study that compared starter versus non-starter players across a full season [13]. The seasonal changes according to the players’ playing time status may help coaching staff to design optimal training programs.

## 2. Materials and Methods

### 2.1. Participants

This study is the second stage in a two-stage investigation conducted on twenty-one professional soccer players (age, 28.3 ± 3.8 yrs; height, 181.2 ± 7.1 cm; and body mass, 74.5 ± 7.7 kg) of one team competing in the Iranian Persian Gulf Pro League were evaluated for 48 weeks during a full season [13]. Previously, the data from the same players were used to determine the differences between starter and non-starter players on weekly TM and TS values across the full soccer season [13]. Specifically, the main focus of this second-stage study is to understand the within-group variations with respect to playing time status. During the full season, there were 44 matches, 200 training sessions, 14,127 min of play and sessions, of which 3960 min are related to participating in match-play; 7 weeks were congested (i.e., two or more matches within seven days), and 30 weeks were non-congested.

The inclusion criteria were to have a minimum three training sessions per week. The two exclusion criteria of this study were (i) the absence of a payer for two weeks, resulting in the player being removed from the study, and (ii) goalkeepers were excluded from the study. Prior to commencing the study, ethical approval was granted from the research ethics committee of the University of Isfahan (IR.UI.REC.1399.064). All players were informed of the purpose of the study before providing signed informed consent. All stages of this study were conducted according to conditions for human studies as outlined in the declaration of Helsinki.

### 2.2. Experimental Approach to the Problem

The study included a full season of a professional soccer team for 48 weeks (W) in the Persian Gulf Pro League and a knockout tournament during the 2018–2019 season. The 48 weeks of the full-season were divided into four periods: pre-season, W1 to W5; early-season, W6 to W19; mid-season, W20 to W35; and end-season, W36 to W48, in order to analyze within-group differences between starting and non-starting players in terms of accelerometer variables obtained using GPS along the season periods.

The criterion for dividing players into two groups based on previous studies was 60 min of play in weekly matches [13,17]. If a competition was not held during a week, the division criterion was based on the total training time for each group per week [13,18,19]. Based on this criterion, 10 players were placed in the starter group, and 11 players were placed in the non-starter group.

### 2.3. External Monitoring Measures

The microelectromechanical system (MEMS) used in this study was a GPSPORTS systems Pty Ltd., model: SPI High-Performance Unit (HPU), made in Australia. It was used in all training and competition sessions during the full season for all players. This tool is based on tracking and SPI HPU and includes GPS position with 15 Hz; Accelerometer: 100 Hz, G Tri-Axial-Track impacts. Mag: 50 Hz, three-axis; Water resistance and data transmission: Infrared and weighs 56 g. The validity and reliability of the device for the variables used in previous studies have been confirmed [20]. A coefficient of variation = 0.90% showed the accuracy of the MEMS used in the present study to track high-sprinting velocities [21].

### 2.4. Data Collection by Wearable Inertial Measurement Unit

Data were exported from the GPS as described by previous studies [13,18,19,22]. The accelerations at zone (AcZ) and decelerations at zone (DcZ) for each level used from the output data for this study are as follows: AcZ1 (<2 m/s^2^); AcZ2 (2 to 4 m/s^2^); AcZ3 (>4 m/s^2^); DcZ1 (<−2 m/s^2^); DcZ2 (−2 to −4 m/s^2^); and DcZ3 (>−4 m/s^2^) [13,23]. Each of these variables was calculated based on acute training load means the total load in a week of body load [24,25]. Afterward, weekly training monotony (wTM = the average weekly acute training load) was divided by the standard deviation of that week [26,27], and the weekly training strain (wTS = obtained from the weekly acute training load multiplying of the wTM) was used [13,27,28].

### 2.5. Statistical Analysis

The Statistical Package for the Social Sciences (SPSS, version 25.0; IBM SPSS Inc., Chicago, IL, USA) was used for statistical procedures and analyses. Data are presented as mean and standard deviation (SD). Kolmogorov–Smirnov and Levene’s tests were executed to check the normality and homogeneity of the data, respectively. Inferential tests were the conducted. Repeated measures analysis of variance (ANOVA) was applied to analyze within-group changes across the different periods of the season for all dependent variables for both starting and non-starting soccer players. Bonferroni post hoc tests were also executed to determine pairwise comparison outcomes. These tests are based on the linearly independent pairwise comparisons among the estimated marginal means. Significant differences were considered for *p* ≤ 0.05. Partial eta squared (ηp^2^) was calculated as the effect size of the repeated measures of ANOVA. Moreover, Hedge’s *g* effect size (95% confidence interval) was calculated to determine the magnitude of the pairwise comparisons. Hopkins’ thresholds for the Cohen d effect size statistics were used [29] as follow: ≤0.2, trivial; >0.2, small; >0.6, moderate; >1.2, large; >2.0, very large; and >4.0, nearly perfect.

## 3. Results

**Figure 1:**
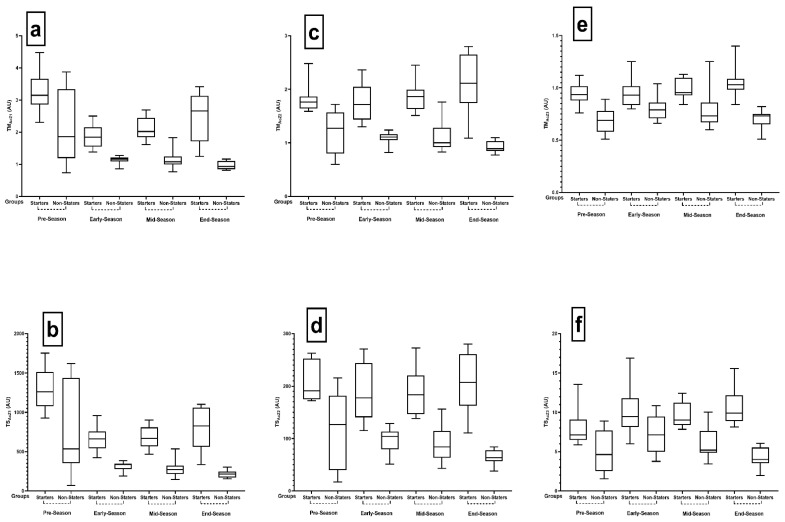
Pairwise comparisons between season periods in TM and TS in all AcZ for both non- and starter players. Abbreviations: TM, training monotony; TS, training strain; AU, arbitrary units; (**a**): TM_AcZ1_, weekly average training monotony based on number of accelerations at Zone 1 (<2 m·s^−2^); (**b**): TS_AcZ1_, weekly average training strain based on number of accelerations at Zone 1 (<2 m·s^−2^); (**c**): TM_AcZ2_, weekly average training monotony based on number of accelerations at Zone 2 (2 to 4 m·s^−2^); (**d**): TS_AcZ2_, weekly average training strain based on number of accelerations at Zone 2 (2 to 4 m·s^−2^); (**e**): TM_AcZ3_, weekly average training monotony based on number of accelerations at Zone 2 (>4 m·s^−2^); (**f**): TS_AcZ3_, weekly average training strain based on number of accelerations at Zone 2 (>4 m·s^−2^).

Intragroup differences for binary comparisons between season periods in TM based on Ac at zone (TM_AcZ_) and TS based on De at zone (TS_DcZ_) and each level for both the starters and non-starters are shown in Table 1, Table 2, Table 3, Table 4, Table 5 and Table 6. Table 1 illustrates the intragroup comparisons of TM_AcZ1_ and TS_AcZ1_ between the different periods of the season for both starters and non-starters. The outcomes revealed significant differences between season periods for starters (*p* < 0.001, ηp^2^ = 0.759) and non-starters (*p* < 0.001, ηp^2^ = 0.393) at TM_AcZ1_ as well as for TS_AcZ1_ between starters (*p* < 0.001, ηp^2^ = 0.717) and non-starters (*p* = 0.029, ηp^2^ = 0.405). The dual sets TM_AcZ1_ and TS_AcZ1_ are shown in Table 1 and Figure 1a,b.

Table 2 and Figure 1c,d present the intragroup comparisons of TM_AcZ2_ and TS_AcZ2_ between the different periods of the season for both the starters and non-starters. According to Table 2, TM_AcZ2_ analysis for the starters (*p* = 0.031, ηp^2^ = 0.397) showed significant results. However, there were no significant changes in TM_AcZ2_ (*p* = 0.280, ηp^2^ = 0.197) in the non-starters nor in TS_AcZ2_ for the starters (*p* = 0.102, ηp^2^ = 0.299) and non-starters (*p* = 0.054, ηp^2^ = 0.354).

Table 3 and Figure 1e,f demonstrate the intragroup comparisons of TM_AcZ3_ and TS_AcZ3_ between the different periods of the season for both starters and non-starters. The results indicated that there were no meaningful differences in the starters and non-starters (*p* = 0.114, ηp^2^ = 0.288 and *p* = 0.095, ηp^2^ = 0.305, respectively). TS_AcZ3_ analysis showed that there were significant differences between the starters and non-starters (*p* = 0.017, ηp^2^ = 0.441 and *p* = 0.019, ηp^2^ = 0.435, respectively).

**Figure 2:**
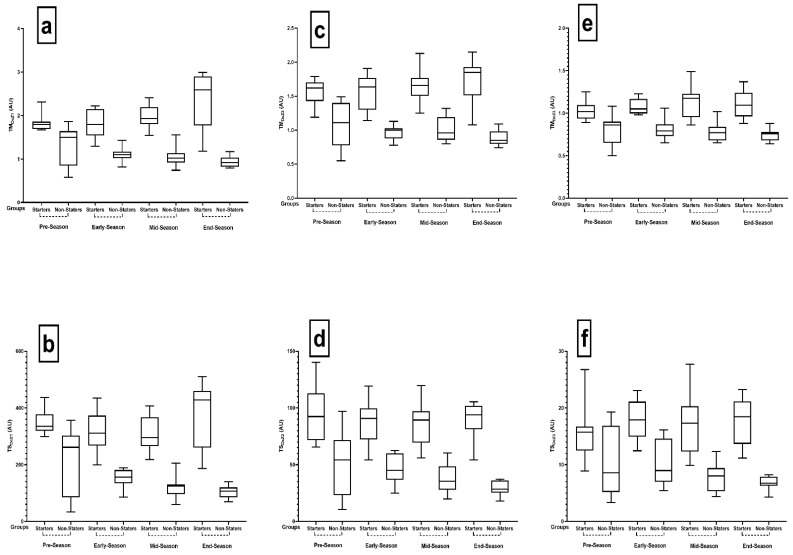
Pairwise comparisons between season periods in TM and TS in all DcZ for both non- and starter players. Abbreviations: TM, training monotony; TS, training strain; AU, arbitrary units; (**a**): TM_AcZ1_, weekly average training monotony based on number of decelerations at Zone 1 (<2 m·s^−2^); (**b**): TS_AcZ1_, weekly average training strain based on number of decelerations at Zone 1 (<2 m·s^−2^); (**c**): TM_AcZ2_, weekly average training monotony based on number of decelerations at Zone 2 (2 to 4 m·s^−2^); (**d**): TS_AcZ2_, weekly average training strain based on number of decelerations at Zone 2 (2 to 4 m·s^−2^); (**e**): TM_AcZ3_, weekly average training monotony based on number of decelerations at Zone 2 (>4 m·s^−2^); (**f**): TS_AcZ3_, weekly average training strain based on number of decelerations at Zone 2 (>4 m·s^−2^).

The intragroup comparisons of TM_DcZ1_ and TS_DcZ1_ between the different periods of the season for both the starters and non-starters are represented in Table 4 and Figure 2a,b. Analysis of TM_DcZ1_ for the starters (*p* = 0.002, ηp^2^ = 0.582) demonstrated meaningful results but did not show significant differences in the non-starters (*p* = 0.136, ηp^2^ = 0.272). TS_DcZ1_ analyses showed that this was significant for both groups (*p* = 0.018, ηp^2^ = 0.437).

The intragroup comparisons of TM_DcZ2_ and TS_DcZ2_ between the different periods of the season for both the starters and non-starters are represented in Table 5 and Figure 1c,d. The results of the TM_DcZ2_ showed that starters had significant changes (*p* = 0.031, ηp^2^ = 0.397), while no changes were observed in the non-starters (*p* = 0.224, ηp^2^ = 0.221). The TS_DcZ2_ workload displayed meaningful changes for the non-starters (*p* = 0.011, ηp^2^ = 0.469), while no changes (*p* = 0.789, ηp^2^ = 0.058) were shown for the starters.

The intragroup comparisons of TM_DcZ3_ and TS_DcZ3_ between the different periods of the season for both the starters and non-starters are represented in Table 6 and Figure 1e,f. There were no significant changes observed in training workload TM_DcZ3_ in any group. However, non-starters (*p* = 0.034, ηp^2^ = 0.392) had significant changes in TS_DcZ3_.

## 4. Discussion

This study is the second part of a previously reported research study [13]. Thus, within-group differences in TM and TS in starters and non-starter soccer players at different speed zones across the periods of the full season were examined. The results showed that both starters and non-starters obtained the highest mean TM and TS scores in the pre-season and the highest accelerations at Zone 1. Moreover, the highest TM and TS scores were exhibited in the end-season and the pre-season decelerations at Zone 1 for starter and non-starter players, respectively. Regardless of match starting status, players had similar TM and TS scores at other speed zones and periods.

To the best of the authors’ knowledge, there is no previous study on in-season intragroup variations in TM and TS among starters and non-starter soccer players at different speed zones. This makes it difficult to compare the results of the present study with those of earlier reports. However, the results are partly in line with the findings of Fessi et al. [11]. The authors of that study investigated changes in several psychophysical parameters such as the stress, affective valence, quantity of perceived internal training load, quality of sleep, fatigue, and muscle soreness of seventeen professional male soccer players during pre- and in-season periods. Their results demonstrated significantly higher monotony and strain values during the pre-season [11]. Similar results were also observed in a recent study by Clemente et al. [30]. In fact, these authors [30] studied the weekly monotony and strain measures throughout a professional soccer season. They monitored nineteen male players for forty-five weeks by using a GPS device. As a result, they noted greater monotony and strain scores during the pre-season compared to scores exhibited during the season. The higher monotony and strain values during the pre-season might be attributed to a higher perception of effort by the players as a consequence of emphasizing technical/tactical elements and greater training intensity during this period [31,32].

Overall, the results revealed that both starter and non-starter players had greater mean monotony and strain values when they performed acceleration and deceleration at speed Zone 1. In other words, there is an increase in TM and TS at slower speed activities. In another study, Fessi et al. [33] examined the influences of reducing training load on TM and TS in professional male soccer players. They compared weekly training load, monotony, strain, and physical performance indicators of players over seventeen standard and seven taper weeks. Consequently, they found lower monotony and strain values in taper weeks compared to standard weeks. It seems that more research is needed to provide a better understanding of the associations between the level of accelerations and decelerations and the psychophysical perceptions of players.

There are some limitations of the present study that should be addressed. The main limitation is that the data were collected from one professional soccer team, and thus, the study was conducted on small sample size. Furthermore, the study lacks information regarding other important parameters such as physical and technical performance indicators and injury prevalence. Additional studies are warranted to analyze the interactions between the measures of training load, functional capacities, and injury incidence in a larger group of players. For example, analyzing possible inverse relationships between accumulating training load and various physical fitness parameters during the different periods of the season in both male and female professional soccer players will be the topic of future research.

## 5. Conclusions

In conclusion, this study is the first to provide information on the intragroup differences in wearable inertial measurement unit derived accelerations, decelerations, and monotony and strain values at different acceleration and deceleration speed zones both starter and non-starter professional soccer players during the different periods of a season. The findings of the study highlighted the variations in TM and TS with regard to the match starting status of players and certain periods of the season. More specifically, regardless of match starting status, at lower acceleration speed zones, the highest mean monotony and strain values were observed during the initial weeks of the season. On the other hand, at lower deceleration speed zones, the highest monotony and strain values were found during the end-season and pre-season for both starter and non-starter players, respectively. Comparisons of within-group values for both starter and non-starter players showed that they had similar monotony and strain values at other speed zones during periods of the season. It is suggested that coaches monitor these two training load indices in order to design optimal individual training programs. Moreover, measuring monotony and strain according to the playing time status of the players may not only help them to facilitate physical and technical development but may also provide valuable information to minimize potential overtraining and injury. Differences in weekly physical loads due to unequal match playing time may be reduced with an adjusted training programs for non-starters.

## Figures and Tables

**Table 1:** Intragroup differences for binary comparisons between season periods in TM and TS in AcZ1 for both non- and starter players.

Variables	Period	Mean (SD)	COMPARATIVE	Groups	*p*	Hedge’s *g* (95% CI)
**TM_AcZ1_ (AU)**	Pre-season	**Starters**: 3.25 ± 0.62 **Non-starters**: 2.18 ± 1.14	PreS vs. EarS	Starters	**0.002**	2.60 [1.41 to 3.79]
				Non-starters	**0.016**	1.24 [0.33 to 2.16]
			PreS vs. MidS	Starters	**0.010**	2.13 [1.03 to 3.23]
	Early-season	**Starters**: 1.87 ± 0.36 **Non-starters**: 1.13 ± 0.13		Non-starters	**0.016**	1.19 [0.28 to 2.10]
			PreS vs. EndS	Starters	0.320	1.04 [0.11 to 1.98]
				Non-starters	**0.020**	1.42 [0.49 to 2.36]
	Mid-season	**Starters**: 2.12 ± 0.37 **Non-starters**: 1.15 ± 0.27	EarS vs. MidS	Starters	0.143	−0.64 [−1.54 to 0.26]
				Non-starters	>0.999	−0.10 [−0.94 to 0.74]
			EarS vs. EndS	Starters	**0.003**	−0.95 [−1.88 to −0.03]
	End-season	**Starters**: 2.48 ± 0.79 **Non-starters**: 0.98 ± 0.13		Non-starters	>0.999	1.15 [0.25 to 2.05]
			MidS vs. EndS	Starters	**0.021**	–0.57 [–1.46 to 0.33]
				Non-starters	0.679	0.78 [–0.09 to 1.65]
**TS_AcZ1_ (AU)**	Pre-season	**Starters**: 1288.97 ± 265.15 **Non-starters**: 765.44 ± 547.51	PreS vs. EarS	Starters	**0.002**	2.76 [1.54 to 3.98]
				Non-starters	**0.022**	1.13 [0.23 to 2.03]
			PreS vs. MidS	Starters	**0.003**	2.68 [1.47 to 3.88]
	Early-season	**Starters**: 659.98 ± 158.04 **Non-starters**: 306.50 ± 63.24		Non-starters	**0.013**	1.18 [0.27 to 2.09]
			PreS vs. EndS	Starters	**0.039**	1.74 [0.71 to 2.76]
				Non-starters	**0.013**	1.36 [0.43 to 2.29]
	Mid-season	**Starters**: 683.62 ± 153.10 **Non-starters**: 282.55 ± 101.83	EarS vs. MidS	Starters	>0.999	–0.15 [–1.02 to 0.73]
				Non-starters	>0.999	0.27 [−0.57 to 1.11]
			EarS vs. EndS	Starters	0.114	−0.56 [−1.45 to 0.33]
	End-season	**Starters**: 793.44 ± 281.32 **Non-starters**: 216.63 ± 47.49		Non-starters	0.515	1.55 [0.59 to 2.50]
			MidS vs. EndS	Starters	**0.047**	−0.46 [−1.35 to 0.42]
				Non-starters	0.463	0.80 [−0.47 to 1.67]

Abbreviations: TM, training monotony; TS, training strain; AU, arbitrary units; PreS, pre-season period; EarS, early-season period; MidS, mid-season period; EndS, end-season period; TM_AcZ1_, weekly average training monotony based on number of accelerations at Zone 1 (<2 m·s^−2^); TS_AcZ1_, weekly average training strain based on number of accelerations at Zone 1 (<2 m·s^−2^); *p*, *p*-value at alpha level 0.05; Hedges’s g (95% CI), Hedges’s g effect size magnitude with 95% confidence interval. Significant differences (*p* ≤ 0.05) are highlighted in bold.

**Table 2:** Intragroup differences for binary comparisons between season periods in TM and TS in AcZ2 for both non- and starter players.

Variables	Period	Mean (SD)	COMPARATIVE	Groups	*p*	Hedge’s *g* (95% CI)
**TM_AcZ2_ (AU)**	Pre-season	**Starters**: 1.82 ± 0.26 **Non-starters**: 1.19 ± 0.39	PreS vs. EarS	Starters	>0.999	0.18 [−0.70 to 1.06]
				Non-starters	>0.999	0.36 [−0.48 to 1.20]
			PreS vs. MidS	Starters	>0.999	−0.20 [−1.08 to 0.68]
	Early-season	**Starters**: 1.76 ± 0.36 **Non-starters**: 1.09 ± 0.12		Non-starters	>0.999	0.24 [−0.59 to 1.08]
			PreS vs. EndS	Starters	0.365	−0.63 [−1.53 to 0.27]
				Non-starters	0.387	0.90 [0.02 to 1.77]
	Mid-season	**Starters**: 1.87 ± 0.27 **Non-starters**: 1.11 ± 0.27	EarS vs. MidS	Starters	0.980	−0.34 [−1.22 to 0.54]
				Non-starters	>0.999	−0.11 [−0.94 to 0.73]
			EarS vs. EndS	Starters	0.013	−0.70 [−1.61 to 0.20]
	End-season	**Starters**: 2.10 ± 0.55 **Non-starters**: 0.93 ± 0.10		Non-starters	0.630	1.32 [0.40 to 2.24]
			MidS vs. EndS	Starters	0.174	−0.51 [−1.40 to 0.39]
				Non-starters	0.389	0.85 [−0.03 to 1.72]
**TS_AcZ2_ (AU)**	Pre-season	**Starters**: 209.71 ± 38.04 **Non-starters**: 112.24 ± 71.12	PreS vs. EarS	Starters	>0.999	0.45 [−0.44 to 1.33]
				Non-starters	>0.999	0.26 [−0.58 to 1.10]
			PreS vs. MidS	Starters	>0.999	0.51 [−0.38 to 1.40]
	Early-season	**Starters**: 187.65 ± 55.12 **Non-starters**: 97.81 ± 23.62		Non-starters	0.704	0.44 [−0.40 to 1.28]
			PreS vs. EndS	Starters	>0.999	0.04 [−0.83 to 0.92]
				Non-starters	0.114	0.89 [0.02 to 1.77]
	Mid-season	**Starters**: 188.62 ± 41.45 **Non-starters**: 87.19 ± 31.90	EarS vs. MidS	Starters	>0.999	−0.02 [−0.90 to 0.86]
				Non-starters	>0.999	0.36 [−0.48 to 1.21]
			EarS vs. EndS	Starters	0.870	−0.35 [−1.23 to 0.54]
	End-season	**Starters**: 207.53 ± 54.59 **Non-starters**: 64.75 ± 12.88		Non-starters	0.094	1.67 [0.70 to 2.64]
			MidS vs. EndS	Starters	0.227	−0.37 [−1.26 to 0.51]
				Non-starters	0.072	0.89 [0.01 to 1.76]

Abbreviations: TM, training monotony; TS, training strain; AU, arbitrary units; PreS, preseason period; EarS, early-season period; MidS, mid-season period; EndS, end-season period; TM_AcZ2_, weekly average training monotony based on number of accelerations at Zone 2 (2 to 4 m·s^−2^); TS_AcZ2_, weekly average training strain based on number of accelerations at Zone 2 (2 to 4 m·s^−2^); *p*, *p*-value at alpha level 0.05; Hedges’s g (95% CI), Hedges’s g effect size magnitude with 95% confidence interval. Significant differences (*p* ≤ 0.05) are highlighted in bold.

**Table 3:** Intragroup differences for binary comparisons between season periods in TM and TS in AcZ3 for both non- and starter players.

Variables	Period	Mean (SD)	COMPARATIVE	Group	*p*	Hedge’s *g* (95% CI)
**TM_AcZ3_ (AU)**	Pre-season	**Starters**: 0.94 ± 0.10 **Non-starters**: 0.70 ± 0.13	PreS vs. EarS	Starters	>0.999	−0.05 [−0.93 to 0.83]
				Non-starters	0.161	−0.84 [−1.74 to 0.03]
			PreS vs. MidS	Starters	>0.999	−0.39 [−1.28 to 0.49]
	Early-season	**Starters**: 0.95 ± 0.14 **Non-starters**: 0.80 ± 0.11		Non-starters	0.678	−0.51 [−1.36 to 0.34]
			PreS vs. EndS	Starters	0.083	−0.83 [−1.74 to 0.08]
				Non-starters	>0.999	−0.03 [−0.87 to 0.80]
	Mid-season	**Starters**: 0.98 ± 0.09 **Non-starters**: 0.78 ± 0.18	EarS vs. MidS	Starters	>0.999	−0.27 [−1.15 to 0.61]
				Non-starters	>0.999	0.13 [−0.70 to 0.97]
			EarS vs. EndS	Starters	0.258	−0.68 [−1.58 to 0.22]
	End-season	**Starters**: 1.05 ± 0.14 **Non-starters**: 0.70 ± 0.09		Non-starters	0.241	0.96 [0.08 to 1.84]
			MidS vs. EndS	Starters	>0.999	−0.53 [−1.42 to 0.36]
				Non-starters	0.767	0.53 [−0.32 to 1.38]
**TS_AcZ3_ (AU)**	Pre-season	**Starters**: 8.09 ± 2.40 **Non-starters**: 4.86 ± 2.59	PreS vs. EarS	Starters	0.158	−0.71 [−1.96 to 0.19]
				Non-starters	0.056	−0.93 [−1.81 to −0.05]
			PreS vs. MidS	Starters	0.221	−0.73 [−1.63 to 0.18]
	Early-season	**Starters**: 10.17 ± 3.14 **Non-starters**: 7.24 ± 2.31		Non-starters	0.432	−0.50 [−1.38 to 0.35]
			PreS vs. EndS	Starters	**0.008**	−1.02 [−1.95 to −0.09]
				Non-starters	>0.999	0.24 [−0.60 to 1.08]
	Mid-season	**Starters**: 9.65 ± 1.60 **Non-starters**: 6.12 ± 2.25	EarS vs. MidS	Starters	>0.999	0.20 −0.68 to 1.08]
				Non-starters	0.857	0.47 [−0.37 to 1.32]
			EarS vs. EndS	Starters	>0.999	−0.15 [−1.03 to 0.72]
	End-season	**Starters**: 10.61 ± 2.31 **Non-starters**: 4.35 ± 1.23		Non-starters	**0.011**	1.50 [0.56 to 2.45]
			MidS vs. EndS	Starters	>0.999	−0.46 [−1.35 to 0.43]
				Non-starters	0.118	0.93 [0.05 to 1.82]

Abbreviations: TM, training monotony; TS, training strain; AU, arbitrary units; PreS, preseason period; EarS, early-season period; MidS, mid-season period; EndS, end-season period; TM_AcZ3_, weekly average training monotony based on number of accelerations at Zone 2 (>4 m·s^−2^); TS_AcZ3_, weekly average training strain based on number of accelerations at Zone 2 (>4 m·s^−2^); *p*, *p*-value at alpha level 0.05; Hedges’s g (95% CI), Hedges’s g effect size magnitude with 95% confidence interval. Significant differences (*p* ≤ 0.05) are highlighted in bold.

**Table 4:** Intragroup differences for binary comparisons between season periods in TM and TS in DcZ1 for both non- and starter players.

Variables	Period	Mean (SD)	COMPARATIVE	Groups	*p*	Hedge’s *g* (95% CI)
**TM_DcZ1_ (AU)**	Pre-season	**Starters**: 1.82 ± 0.19 **Non-starters**: 1.31 ± 0.44	PreS vs. EarS	Starters	>0.999	0.07 [−0.81 to 0.95]
				Non-starters	0.626	0.64 [−0.22 to 1.50]
			PreS vs. MidS	Starters	>0.999	−0.65 [−1.55 to 0.25]
	Early-season	**Starters**: 1.80 ± 0.33 **Non-starters**: 1.09 ± 0.16		Non-starters	0.176	0.68 [−0.18 to 1.54]
			PreS vs. EndS	Starters	**0.045**	−0.99 [−1.98 to −0.06]
				Non-starters	0.202	1.10 [0.21 to 2.00]
	Mid-season	**Starters**: 1.98 ± 0.27 **Non-starters**: 1.06 ± 0.22	EarS vs. MidS	Starters	0.161	−0.57 [−1.46 to 0.33]
				Non-starters	>0.999	0.14 [–0.70 to 0.97]
			EarS vs. EndS	Starters	**0.001**	−0.96 [−1.88 to −0.03]
	End-season	**Starters**: 2.33 ± 0.67 **Non-starters**: 0.94 ± 0.12		Non-starters	0.966	1.03 [0.14 to 1.92]
			MidS vs. EndS	Starters	**0.042**	−0.66 [−1.56 to 0.26]
				Non-starters	>0.999	0.67 [−0.19 to 1.53]
**TS_DcZ1_ (AU)**	Pre-season	**Starters**: 349.37 ± 42.58 **Non-starters**: 208.15 ± 116.22	PreS vs. EarS	Starters	>0.999	0.54 [−0.35 to 1.43]
				Non-starters	0.415	0.64 [−0.22 to 1.49]
			PreS vs. MidS	Starters	>0.999	0.69 [−0.21 to 1.60]
	Early-season	**Starters**: 315.25 ± 74.37 **Non-starters**: 151.32 ± 35.13		Non-starters	**0.030**	0.95 [0.07 to 1.83]
			PreS vs. EndS	Starters	>0.999	−0.26 [−1.15 to 0.62]
				Non-starters	**0.023**	1.21 [0.30 to 2.12]
	Mid-season	**Starters**: 310.75 ± 62.22 **Non-starters**: 122.72 ± 38.47	EarS vs. MidS	Starters	>0.999	0.06 [−0.81 to 0.94]
				Non-starters	0.591	0.75 [−0.12 to 1.61]
			EarS vs. EndS	Starters	0.059	−0.57 [−1.47 to 0.32]
	End-season	**Starters**: 373.32 ± 114.88 **Non-starters**: 103.09 ± 22.17		Non-starters	0.132	1.58 [0.62 to 2.54]
			MidS vs. EndS	Starters	**0.018**	−0.65 [−1.54 to 0.25]
				Non-starters	>0.999	0.60 [−0.25 to 1.46]

Abbreviations: TM, training monotony; TS, training strain; AU, arbitrary units; PreS, preseason period; EarS, early-season period; MidS, mid-season period; EndS, end-season period; TM_AcZ1_, weekly average training monotony based on number of decelerations at Zone 1 (<−2 m·s^−2^); TS_AcZ1_, weekly average training strain based on number of decelerations at Zone 1 (<−2 m·s^−2^); *p*, *p*-value at alpha level 0.05; Hedges’s g (95% CI), Hedges’s g effect size magnitude with 95% confidence interval. Significant differences (*p* ≤ 0.05) are highlighted in bold.

**Table 5:** Intragroup differences for binary comparisons between season periods in TM and TS in DcZ2 for both non- and starter players.

Variables	Period	Mean (SD)	COMPARATIVE	Groups	*p*	Hedge’s *g* (95% CI)
**TM_DcZ2_ (AU)**	Pre-season	**Starters**: 1.56 ± 0.19 **Non-starters**: 1.07 ± 0.32	PreS vs. EarS	Starters	<0.999	0.03 [−0.85 to 0.91]
				Non-starters	<0.999	0.40 [–0.44 to 1.24]
			PreS vs. MidS	Starters	<0.999	–0.38 [–1.26 to 0.50]
	Early-season	**Starters**: 1.56 ± 0.26 **Non-starters**: 0.97 ± 0.11		Non-starters	<0.999	0.22 [–0.62 to 1.05]
			PreS vs. EndS	Starters	0.467	–0.60 [–1.50 to 0.30]
				Non-starters	0.238	0.77 [–0.09 to 1.64]
	Mid-season	**Starters**: 1.65 ± 0.24 **Non-starters**: 1.01 ± 0.17	EarS vs. MidS	Starters	0.725	–0.35 [–1.24 to 0.53]
				Non-starters	<0.999	–0.27 [–1.11 to 0.57]
			EarS vs. EndS	Starters	0.093	–0.57 [–1.46 to 0.33]
	End-season	**Starters**: 1.73 ± 0.33 **Non-starters**: 0.88 ± 0.12		Non-starters	0.942	0.78 [−0.08 to 1.65]
			MidS vs. EndS	Starters	<0.999	−0.28 [−1.16 to 0.60]
				Non-starters	0.561	0.87 [−0.001 to 1.75]
**TS_DcZ2_ (AU)**	Pre-season	**Starters**: 94.91 ± 24.53 **Non-starters**: 50.88 ± 29.47	PreS vs. EarS	Starters	<0.999	0.32 [−0.56 to 1.21]
				Non-starters	<0.999	0.24 [−0.60 to 1.08]
			PreS vs. MidS	Starters	<0.999	0.39 [−0.50 to 1.27]
	Early-season	**Starters**: 87.25 ± 20.56 **Non-starters**: 45.25 ± 13.33		Non-starters	0.795	0.53 [−0.32 to 1.38]
			PreS vs. EndS	Starters	<0.999	0.26 [−0.62 to 1.14]
				Non-starters	0.055	0.99 [0.10 to 1.87]
	Mid-season	**Starters**: 86.08 ± 18.99 **Non-starters**: 38.33 ± 12.96	EarS vs. MidS	Starters	<0.999	0.06 [−0.82 to 0.93]
				Non-starters	0.655	0.51 [−0.34 to 1.36]
			EarS vs. EndS	Starters	<0.999	−0.10 [−0.97 to 0.78]
	End-season	**Starters**: 89.15 ± 17.18 **Non-starters**: 28.96 ± 6.29		Non-starters	**0.019**	1.50 [0.56 to 2.45]
			MidS vs. EndS	Starters	<0.999	−0.16 [−1.04 to 0.72]
				Non-starters	0.332	0.88 [0.01 to 1.76]

Abbreviations: TM, training monotony; TS, training strain; AU, arbitrary units; PreS, preseason period; EarS, early-season period; MidS, mid-season period; EndS, end-season period; TM_AcZ2_, weekly average training monotony based on number of decelerations at Zone 2 (−2 to −4 m·s^−2^); TS_AcZ2_, weekly average training strain based on number of decelerations at Zone 2 (−2 to −4 m·s^−2^); *p*, *p*-value at alpha level 0.05; Hedges’s g (95% CI), Hedges’s g effect size magnitude with 95% confidence interval. Significant differences (*p* ≤ 0.05) are highlighted in bold.

**Table 6:** Intragroup differences for binary comparisons between season periods in TM and TS in DcZ3 for both non- and starter players.

Variables	Period	Mean (SD)	COMPARATIVE	Group	*p*	Hedge’s *g* (95% CI)
**TM_DcZ3_ (AU)**	Pre-season	**Starters**: 1.03 ± 0.11 **Non-starters**: 0.80 ± 0.18	PreS vs. EarS	Starters	<0.999	−0.42 [−1.30 to 0.47]
				Non-starters	<0.999	−0.04 [−0.88 to 0.80]
			PreS vs. MidS	Starters	0.357	−0.69 [−1.59 to 0.21]
	Early-season	**Starters**: 1.08 ± 0.09 **Non-starters**: 0.81 ± 0.12		Non-starters	<0.999	0.13 [−0.70 to 0.97]
			PreS vs. EndS	Starters	0.652	−0.56 [−1.45 to 0.34]
				Non-starters	<0.999	0.41 [−0.44 to 1.25]
	Mid-season	**Starters**: 1.14 ± 0.19 **Non-starters**: 0.78 ± 0.11	EarS vs. MidS	Starters	0.960	−0.43 [−1.32 to 0.45]
				Non-starters	<0.999	0.23 [−0.61 to 1.06]
			EarS vs. EndS	Starters	<0.999	−0.26 [−1.14 to 0.62]
	End-season	**Starters**: 1.11 ± 0.16 **Non-starters**: 0.74 ± 0.07		Non-starters	0.726	0.64 [−0.22 to 1.50]
			MidS vs. EndS	Starters	<0.999	0.18 [−0.70 to 1.05]
				Non-starters	<0.999	0.38 [−0.47 to 1.22]
**TS_DcZ3_ (AU)**	Pre-season	**Starters**: 15.76 ± 4.63 **Non-starters**: 10.77 ± 5.99	PreS vs. EarS	Starters	<0.999	−0.50 [−1.39 to 0.39]
				Non-starters	<0.999	0.03 [−0.81 to 0.86]
			PreS vs. MidS	Starters	<0.999	−0.31 [−1.19 to 0.57]
	Early-season	**Starters**: 17.88 ± 3.47 **Non-starters**: 10.64 ± 4.03		Non-starters	0.917	0.58 [−0.27 to 1.44]
			PreS vs. EndS	Starters	<0.999	−0.35 [−1.23 to 0.53]
				Non-starters	0.180	0.89 [0.01 to 1.76]
	Mid-season	**Starters**: 17.36 ± 5.33 **Non-starters**: 7.98 ± 2.54	EarS vs. MidS	Starters	<0.999	0.11 [−0.77 to 0.99]
				Non-starters	0.562	0.76 [−0.11 to 1.62]
			EarS vs. EndS	Starters	<0.999	0.12 [−0.75 to 1.00]
	End-season	**Starters**: 17.38 ± 4.22 **Non-starters**: 6.77 ± 1.30		Non-starters	**0.026**	1.24 [0.33 to 2.15]
			MidS vs. EndS	Starters	<0.999	−0.004 [−0.88 to 0.87]
				Non-starters	<0.999	0.58 [−0.28 to 1.43]

Abbreviations: TM, training monotony; TS, training strain; AU, arbitrary units; PreS, preseason period; EarS, early-season period; MidS, mid-season period; EndS, end-season period; TM_AcZ3_, weekly average training monotony based on number of decelerations at Zone 2 (>−4 m·s^−2^); TS_AcZ3_, weekly average training strain based on number of decelerations at Zone 2 (>−4 m·s^−2^); *p*, *p*-value at alpha level 0.05; Hedges’s g (95% CI), Hedges’s g effect size magnitude with 95% confidence interval. Significant differences (*p* ≤ 0.05) are highlighted in bold.

## Data Availability

The datasets used and/or analyzed during the current study are available from the corresponding author on reasonable request.
